# IMPACT: interpretable microbial phenotype analysis via microbial characteristic traits

**DOI:** 10.1093/bioinformatics/btae702

**Published:** 2024-12-10

**Authors:** Daniel Mechtersheimer, Wenze Ding, Xiangnan Xu, Sanghyun Kim, Carolyn Sue, Yue Cao, Jean Yang

**Affiliations:** School of Mathematics and Statistics, The University of Sydney, Sydney, NSW 2006, Australia; Sydney Precision Data Science Centre, The University of Sydney, Sydney, NSW 2006, Australia; Charles Perkins Centre, The University of Sydney, Sydney, NSW 2006, Australia; School of Mathematics and Statistics, The University of Sydney, Sydney, NSW 2006, Australia; Sydney Precision Data Science Centre, The University of Sydney, Sydney, NSW 2006, Australia; Charles Perkins Centre, The University of Sydney, Sydney, NSW 2006, Australia; School of Mathematics and Statistics, The University of Sydney, Sydney, NSW 2006, Australia; Sydney Precision Data Science Centre, The University of Sydney, Sydney, NSW 2006, Australia; Neuroscience Research Australia, Randwick, NSW 2031, Australia; School of Mathematics and Statistics, The University of Sydney, Sydney, NSW 2006, Australia; Sydney Precision Data Science Centre, The University of Sydney, Sydney, NSW 2006, Australia; Charles Perkins Centre, The University of Sydney, Sydney, NSW 2006, Australia; Charles Perkins Centre, The University of Sydney, Sydney, NSW 2006, Australia; Neuroscience Research Australia, Randwick, NSW 2031, Australia; School of Mathematics and Statistics, The University of Sydney, Sydney, NSW 2006, Australia; Sydney Precision Data Science Centre, The University of Sydney, Sydney, NSW 2006, Australia; Charles Perkins Centre, The University of Sydney, Sydney, NSW 2006, Australia; Laboratory of Data Discovery for Health Limited (D24H), New Territories, Hong Kong SAR, China; School of Mathematics and Statistics, The University of Sydney, Sydney, NSW 2006, Australia; Sydney Precision Data Science Centre, The University of Sydney, Sydney, NSW 2006, Australia; Charles Perkins Centre, The University of Sydney, Sydney, NSW 2006, Australia; Laboratory of Data Discovery for Health Limited (D24H), New Territories, Hong Kong SAR, China

## Abstract

**Motivation:**

The human gut microbiome, consisting of trillions of bacteria, significantly impacts health and disease. High-throughput profiling through the advancement of modern technology provides the potential to enhance our understanding of the link between the microbiome and complex disease outcomes. However, there remains an open challenge where current microbiome models lack interpretability of microbial features, limiting a deeper understanding of the role of the gut microbiome in disease. To address this, we present a framework that combines a feature engineering step to transform tabular abundance data to image format using functional microbial annotation databases, with a residual spatial attention transformer block architecture for phenotype classification.

**Results:**

Our model, IMPACT, delivers improved predictive accuracy performance across multiclass classification compared to similar methods. More importantly, our approach provides interpretable feature importance through image classification saliency methods. This enables the extraction of taxa markers (features) associated with a disease outcome and also their associated functional microbial traits and metabolites.

**Availability and implementation:**

IMPACT is available at https://github.com/SydneyBioX/IMPACT. We providedirect installation of IMPACT via pip.

## 1 Introduction

The human body is host to millions of microorganisms that create complex microbial communities, which together are called the human microbiome. Mounting evidence has emerged linking the human microbiome as an important factor in an individual’s health outcomes (Cho and Blaser 2012). More specifically, the gut microbiome, which is the largest microbial community in humans, has been linked to diseases, such as obesity (Castaner *et al.* 2018), Crohn’s disease, cancer therapy responses (Gopalakrishnan *et al.* 2018), cardiovascular disease (Peng *et al.* 2018), and neurological diseases such Alzheimer’s and Parkinson’s disease (PD) (Loh *et al.* 2024). Thus, understanding how the gut microbiome influences host health (Shreiner *et al.* 2015, Singh *et al.* 2017) holds promise for the development of personalized therapies to improve individual health outcomes.

In recent years, deep learning methods have shown to be effective in a variety of health and bioinformatics problems including protein structure prediction (Senior *et al.* 2020), medical imaging, electronic health record analysis (Esteva *et al.* 2019), and more. More recently, a number of approaches have embraced developments in the new field of deep learning and adapted it to improve disease prediction in microbiome data (Chowdhury and Fong 2020, Zhu *et al.* 2020). To date, a number of methods use a 1-dimensional (1D) convolutional neural network (CNN, p × 1) architecture where a number of methods are used to group taxa that give similar information together, such that each node in the CNN extracts similar information. For example, MDeep (Wang *et al.* 2020) use a network architecture that seeks to mimic the structure of a phylogenetic tree for prediction of clinical outcomes. Sharma and colleagues (Sharma *et al.* 2020, Sharma and Xu 2021) used a biologically informed deep learning (DL) approach where inputs are separated and grouped based on their phylum level taxonomic profiles and are fed into 1D CNNs called TaxoNN for phenotype prediction. Other varieties of autoencoder (AE) architectures (Oh and Zhang 2020) were used to provide an alternative approach for grouping similar taxa and performing dimension reduction of the microbiome data.

All these approaches lack interpretability in different ways. For AE architectures, the process of encoding the vector of inputs into a lower dimensional vector given as the output of the encoder removes interpretability as there is no way of quantifying which of the inputs contribute to which of the encoders outputs. Together, all these approaches often lack emphasis on feature selection and do not provide clear pathways to understand taxonomic features based on their association with disease outcomes.

A recent work PopPhy-CNN aims to increase interpretability through the use of a deep learning architecture in which data is transformed to two-dimensional embedding using a phylogenetic relationship at genus level resolution (Reiman *et al.* 2020). While this approach retains a biologically driven approach to taxa similarities and allows for the extraction of feature importance, PopPhy-CNN is limited to examination at genus resolution and does not consider microbial features. To advance our understanding of the role of the gut microbiome the gut microbiome should be viewed not only from the lens of individual taxa, but the functional role these taxa play.

To this end, we propose IMPACT, an interpretable workflow for microbiome data that integrates a unique feature engineering approach delivering robust low-dimensional taxa similarity measures based on functional biological annotations, combined with a deep learning architecture that integrates spatial attention maps into residual blocks. We show our model provides improved phenotype prediction while allowing for interpretable taxa importance at species level based on functional microbial annotations.

## 2 Materials and methods

### 2.1 Data

We examined seven different data sets of PD individuals collected by our team (Supplementary Table S1). Each dataset contains a varying number of healthy controls (HC) and PD individuals. All data were measured from the v3-v4 regions using a 16srRNA sequencing method (Supplementary Fig. S1). These seven datasets were from four different countries, the USA, China, Finland, and Australia. All datasets are publicly available for download from https://sydneybiox.github.io/PD16SData.

We also used the American Gut Dataset in our evaluation (McDonald *et al.* 2018). The American Gut Dataset is a large, open-source collection of gut microbiome data from the American Gut Project. It includes bacterial composition from stool samples and metadata on participants’ health, diet, and lifestyle, used to study the connections between the microbiome and various health outcomes.

We simulated human gut microbial feature abundances using metaSPARSim (Patuzzi *et al.* 2019). MetaSPARSim simulates microbiome count data using a Multivariate Hypergeometric distribution to reflect the sparsity and the composition nature of the microbiome data. We generated a total of 36 microbiome simulation datasets. See Supplementary Methods for details on simulation data and data processing.

### 2.2 Feature engineering

We utilize taxa relative abundance and associated metabolites to produce low-dimensional representations of their similarity by converting non-image, vectorized data into a well-organized image form. Inspired by DeepInsight (Sharma *et al.* 2019), this is implemented through a convex hull algorithm aiming to find the minimum box covering all the sample points after they are embedded into a two-dimensional plane using non-linear dimensionality reduction techniques such as t-distributed stochastic neighbor embedding (t-SNE) (Zhou and Jin 2020) and/or uniform manifold approximation and projection (UMAP) (McInnes *et al.* 2018). This results in a single channel image with similarity between taxa associated with the spatial proximity of the pixels they are assigned to. See Supplementary Methods for more details on feature engineering.

### 2.3 Model framework and architecture

An important aspect of our model is an emphasis on extracting the most accurate representation of which areas of sample images lead to a classification result. To do this, we utilize a residual block architecture which has shown to be especially efficient for training generalizable networks (He *et al.* 2020) which is key given the small sample size of most microbiome datasets.

As to our network architecture, each residual block contains two convolutional layers, with a sequential layer structure as follows: convolutional layer, batch-normalization, activation function, convolutional layer, batch-normalization, spatial attention map, Identity function, and activation function.

This architecture follows the internal sequential structure of residual blocks introduced by the original authors in Deep Residual Learning for Image Recognition (He *et al.* 2015). Our model allows the user to choose the number of filters, filter size, and stride of each residual block explicitly using a simple list structure.

For classification tasks, this methodology utilizes the gradients of classification scores with respect to convolutional layers feature maps to identify the parts of the input image that contribute most to the final classification scores. Typically, Grad-CAM is used to identify which regions of a single sample image lead to a particular classification outcome.

This differs to the pseudo images as all images have the same taxa mapped to the same pixel in every image, the benefit of this is that we can investigate the regions of images which leads to different label classification outcomes across all samples rather than just a single sample to determine which Taxon contributed most to each classification label. Once regions that represent importance have been determined, we can translate this to feature importance based on which features are mapped to regions with high importance.

### 2.4 Performance and evaluation

To compare predictive performance of the model, all seven PD datasets from four different regions were used together as a single dataset. We considered two different classification problems across three evaluation schemes: (i) binary classification of PD status using cross-validation on all datasets; (ii) multiclass classification problem predicting both the PD status and the country of origin for the sample; and (iii) binary classification of PD on independent test sets.

We compared models specially designed for microbiome as well as classical machine learning. These are: (i) TaxoNN using the best-performing architecture as stated in the original paper; (ii) support vector machine; (iii) random forest; and (iv) DeepMicro.

## 3 Results

### 3.1 Integrating taxa similarity and functional traits into interpretable and scalable deep learning models for microbiome data

The goal of IMPACT is to facilitate an interpretable deep-learning model for microbiome data. The key innovation of IMPACT is transforming traditional tabular microbiome data into images, allowing the use of advanced computer vision techniques to perform disease classification and gain insights into important taxa. The three key concepts behind this framework are (i) taxa similarity, (ii) low-dimensional representations, and (iii) image transformation.

The first step involves assessing taxa similarity. IMPACT considers taxa similarity by using functional information of the human cometabolism at species level extracted from the microbial annotation databases Agora2 (Heinken *et al.* 2023) and the Microbe Directory (Shaaban *et al.* 2018). The constructed similarity then helps to reduce the relative abundance matrix into a low-dimensional embedding and transform it into an image format as the input into the deep learning model. We compared this metabolic approach with other common similarities, such as Pearson correlation, phylogeny-based similarity (Cophenetic distance from phylogenetic tree), and Spearman correlation. As shown in Supplementary Fig. S3, all four kinds of similarities contribute to the performance of IMPACT at almost the same level, but our metabolic approach is the most computationally efficient one (directly loading from the Agora2 database without further computation).

**Figure 1.**
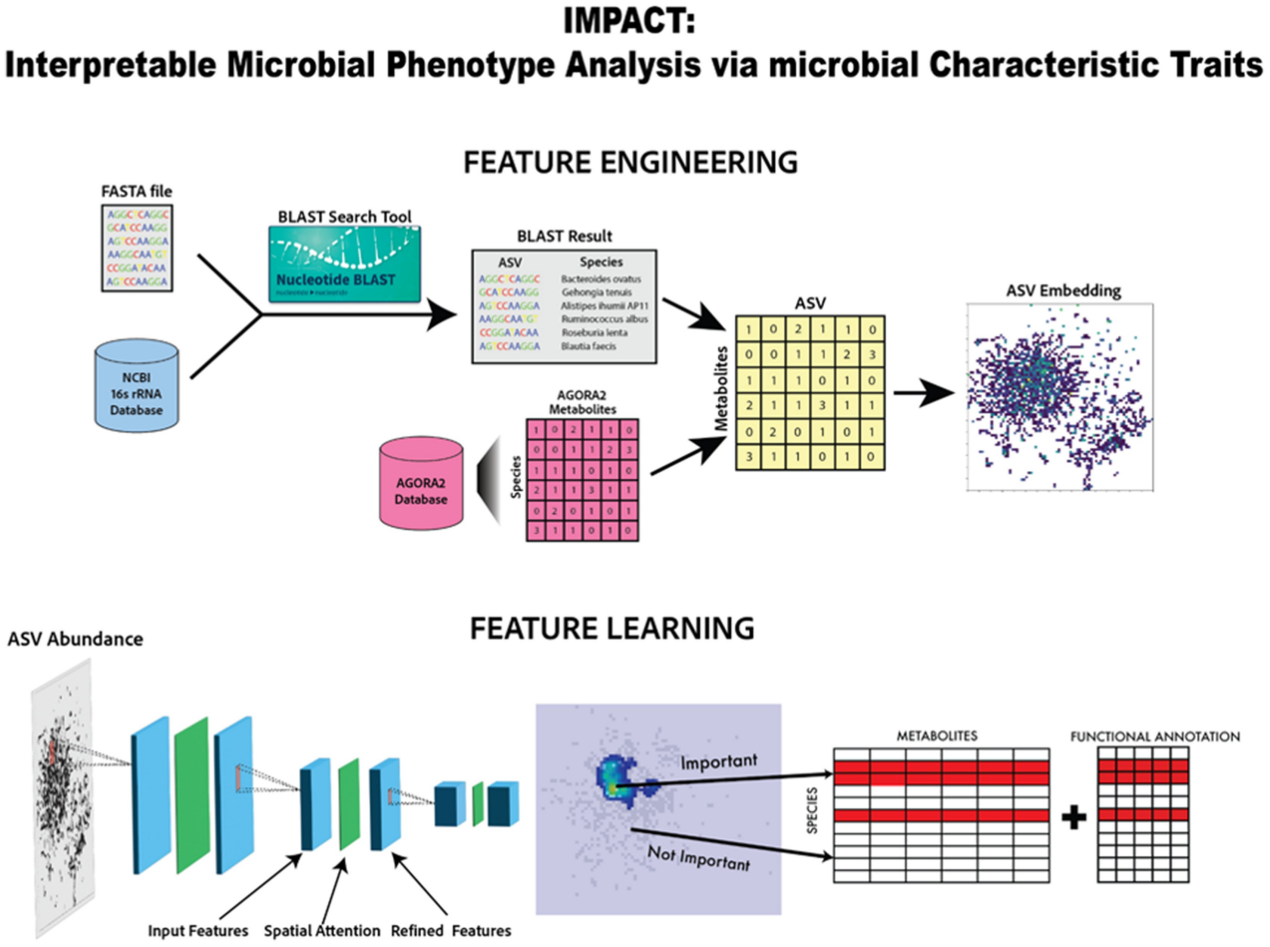
Overview of IMPACT. Schematic overview of IMPACT feature engineering and feature learning to obtain feature importance for species considering metabolite and functional annotation information.

Figure 1 shows the feature engineering process which uses relative abundance and metabolite production of taxa as a reference for spatial taxa similarity in low-dimensional embedding used to generate images. We leverage a CNN architecture that uses residual blocks with built-in spatial attention maps to learn spatially important regions of these images. As all samples have the same two-dimensional embedding of taxa, taxa importance is determined by averaging pixel feature importance extracted with standard computer vision saliency methods. Once taxa importance has been extracted we correlate such information with microbial databases to investigate patterns in metabolites and functional microbial traits such as gram status of important taxa.

### 3.2 IMPACT demonstrates high accuracy in disease phenotype classification

**Figure 2.**
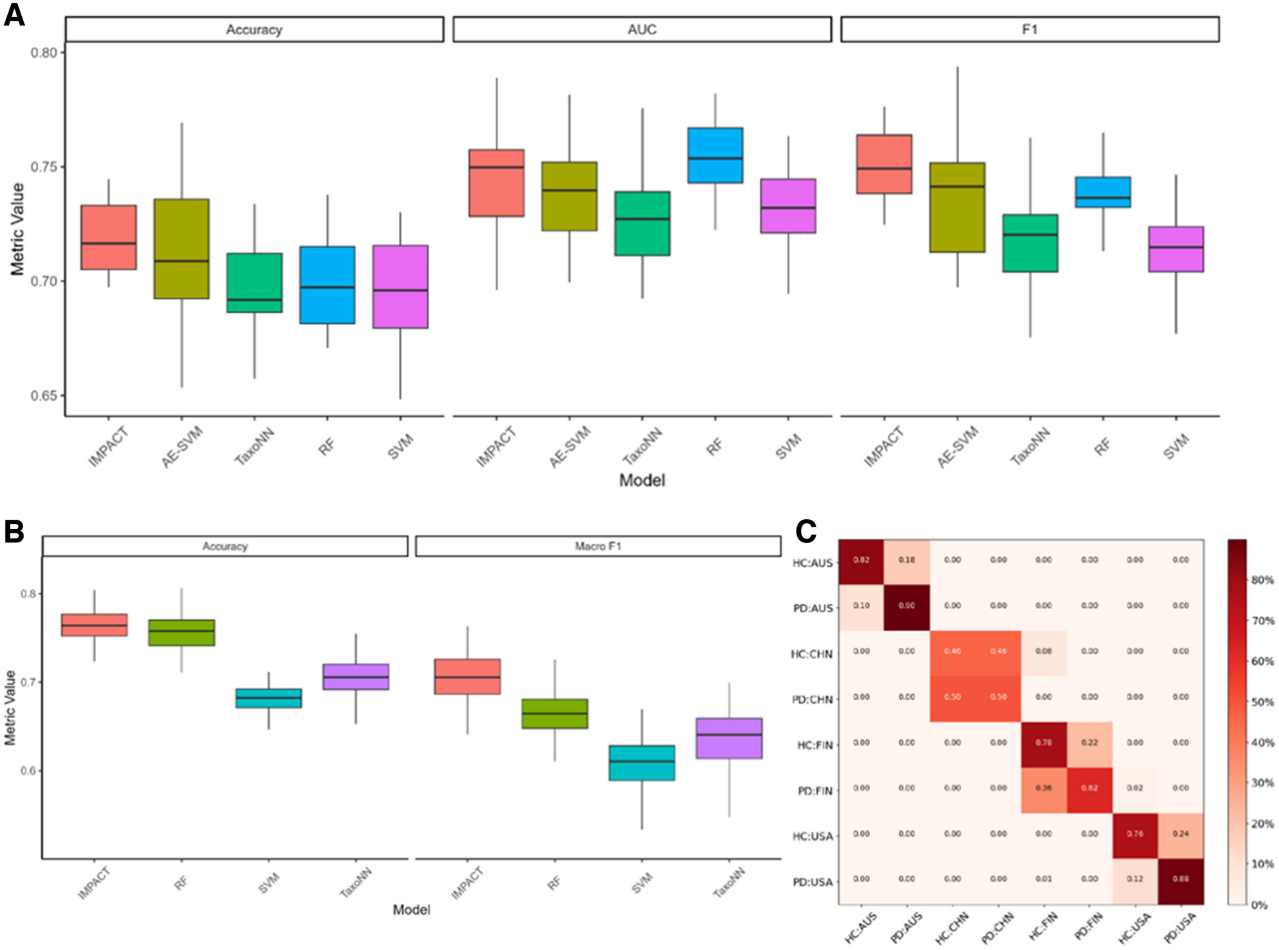
Performance comparison of IMPACT and four other methods. (A) The accuracy, AUC, and FI for biliary disease outcome prediction for five different models. (B) Multiclass (disease plus region) accuracy and Macro F1. (C) Confusion matrix of multiclass label predictions by region and disease status.

To determine the performance of our IMPACT model, we first illustrate an exemplar dataset, the Lubomski data, using PD phenotype classification including and excluding geographic cohort label. We compared the model performance against similar Deep Learning methods TaxoNN (Sharma *et al.* 2020) and DeepMicro (Oh and Zhang 2020) (AE-SVM) and classical machine learning methods, Support Vector Machine (SVM), and Random Forest (RF) using repeated 5-fold cross-validation. Figure 2 shows all aforementioned methods, for DeepMicro we only show the best-performing combination between convolutional AE, AE, variational AE dimension reduction and multilayer perceptron, RF and SVM for classification. We observe in Fig. 2A that IMPACT and DeepMicro both perform well on accuracy compared to TaxoNN, SVM, and RF however RF achieves the highest Area Under the Curve (AUC). For multiclass classification, we observe that IMPACT and RF tend to perform the best for Accuracy and Mean F1 score. Compared with other deep learning methods, IMPACT has two distinct differences, one is the incorporation of metabolic functional information of each taxa, and the other one is the transformation of microbial feature to image. These results showed that such strategies could benefit the useful feature extraction in deep learning models thus improving the prediction.

We also used a large-scale AmericanGut dataset to further validate our method. To enhance the significance of our method, we predicted three various outcomes other than PD and HC, i.e., antibiotic history, patient sex, and age via IMPACT and compared the results with other baseline approaches such as TaxoNN, support vector machine (SVM), and random forest (RF). As shown in Supplementary Fig. S4, IMPACT exhibits generalization ability regarding various outcomes and shows its superiority to other baselines. For example, the average accuracy for sex prediction of IMPACT to others is around 71% versus 49% (random forest), 50% (support vector machine), and 48% (TaxoNN). These results indicate IMPACT could capture the general pattern of experimental gut microbiota data instead of overfitting to specific label-leaded local optimal points. Furthermore, generalization and superiority of IMPACT are not affected by hyper-parameter changes, such as BLAST threshold setting during the data pre-processing. We changed the BLAST threshold from 70% base pair match to 90% and found that IMPACT performs robustly no matter of sex, age, or antibiotic history prediction (Supplementary Fig. S4). More interestingly, other methods all showed the same characteristic when raising the BLAST threshold. Together with the various output validation, we could conclude that the fundamental patterns of microbiota data are stable when such hyper-parameters change.

To further assess the stability of IMPACT, we simulated datasets under various scenarios using parameters estimated from the Aho data of the PD datasets (Supplementary Figs S5–S9). We observed IMPACT is able to discriminate with a fold change of as low as 1.3–1.5, with as few as 45 DA taxa and with <200 total number of taxa. As expected, performance increased as we increased fold change, number of DA taxa, and total number of taxa. Whilst increasing sparsity, that is, when there is less signal in data, led to decrease in performance as expected, the model remained stable across datasets with sparsity levels of 0.78, 0.84, and 0.89, suggesting the model is robust to certain drops in sparsity. In terms of number of samples, IMPACT achieved reliable performance with at least 40 samples in a class.

### 3.3 IMPACT is robust to variation in image representation

**Figure 3.**
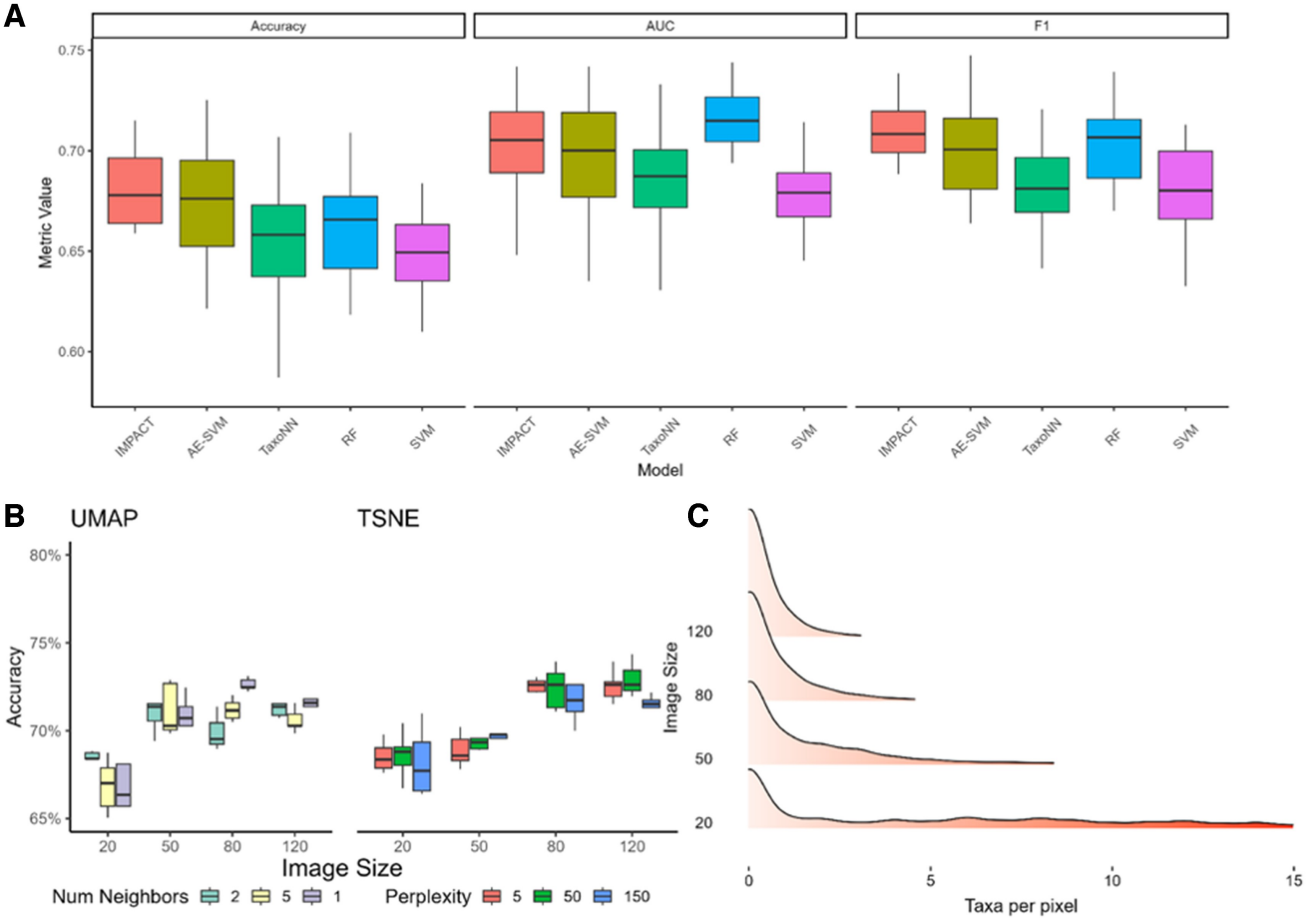
Robustness of the models to varying image properties. (A) Independent test accuracy, AUC, and F1 score set using the Jin dataset. (B) Accuracy for different parameters of UMAP and TSNE two-dimensional embeddings and different image sizes. (C) Distribution of the number of taxa assigned per pixel.

To determine if our model is sensitive to the two-dimensional representations of our features by observing model performance across different representations. We change the t-SNE parameter values for perplexity from 550 100 and number of neighbors from 2515 UMAP. Figure 3 shows no significant changes in terms of accuracy across local versus global structure. Additionally, we do not observe any significant differences in performance when comparing embeddings produced using the same input parameters showing our model is able to achieve the same results and extract the same feature importance making it insensitive to the randomness in the dimension reduction embedding of TSNE and UMAP. That is, parameters that affect dimension reduction do not significantly impact the models ability to learn the relationships in the data, as such it is insensitive to changes in these parameters.

Similar investigation is also done to determine how variations in image size may affect our model performance and interpretability. We use t-SNE with a fixed perplexity value and a fixed random state value to obtain a reproducible embedding result. Typically, there is a concern with computational feasibility with large image sizes however, accuracy reduces with reduced sizes. In other words, to consider as more features will be mapped to the same pixel for smaller images which makes it more challenging to identify the important taxa for a given pixel. Figure 3B shows that performance increases initially as image size increases, as expected. However, beyond a certain value, there appears to be a diminishing return to increasing accuracy. Figure 3C shows for smaller image sizes, up to 15 taxa may be placed in the pixel of an image meaning our model loses the ability to differentiate between the relative abundance values of these taxa.

### 3.4 Geographic variability has minimal impact on PD classification

Current literature shows that gut microbiome compositions tend to be more similar within geographic regions (Yatsunenko *et al.* 2012, Gaulke and Sharpton 2018, He *et al.* 2018). As we have seven datasets from four distinct geographic regions, we have also investigated a multiclass classification framework that predicts phenotype outcome and observed that misclassification occurs primarily for PD status, however, misclassification occurs almost exclusively within the same geographic cohort.

This demonstrates the strong biological signal associated with the unique compositions of different geographic regions based on demographic factors like diet. This result highlights the importance of considering more information than just taxa. The strong signal from geographic regions complicates understanding how the gut contributes to disease outcomes as many taxa may play the same role in disease but only some may be present in a given cohort.

### 3.5 Model interpretability facilitates the identification of features associated with PD phenotype

**Figure 4.**
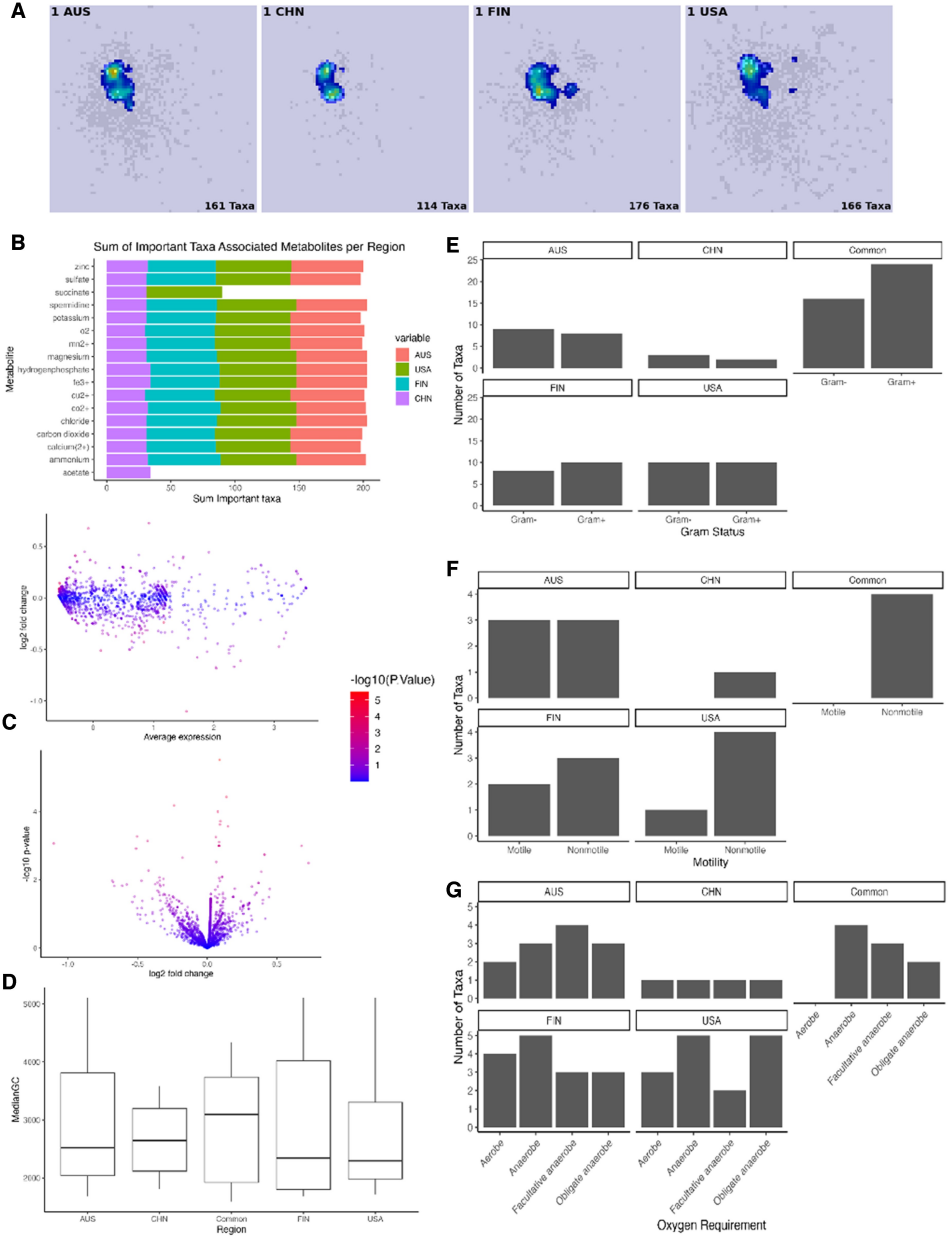
Analysis of the important taxa and associated metabolites across regions. (A) Saliency maps of PD samples using GradCam. (B) Summing across associated metabolites by region and showing only 99th percentile sums by region. (C) Differential expression of metabolites between all important taxa and all unimportant taxa using Volcano and MA plot. (D–G) Functional microbial annotations of important taxa split into taxa unique to each of the four specific regions and important taxa common to all regions.

To facilitate a deeper understanding of similarities between taxa considered important to phenotype outcome, we aim to identify patterns between “important” and “unimportant” taxa based on their associated metabolites and functional microbial features using Agora2 and NCBI databases. Figure 4A shows the saliency maps for PD samples in each region representing the averaging feature importance across all PD samples for a specific region. Figure 4B highlights common metabolites across all regions. The most common metabolite Zinc, has been repeatedly linked to PD (Forsleff *et al.* 1999, Sikora and Ouagazzal 2021) as well as other metal ions like Copper and Iron which appear slightly further down the Fig. 4B and corresponding differential expression analysis of metabolite are presented in Fig. 4C and Supplementary Table S2.

Figure 4D displays functional microbial annotations of key taxa, categorized by whether they are common or regional specific taxa. We observe among common taxa, gram-positive species outnumber gram-negative, but this observation does not hold for region-specific taxa. Similarly, only non-motile taxa are common, while region-specific taxa show both motile and non-motile varieties. While there are no universally common anaerobic taxa, each region’s unique taxa includes them. Notably, the median gene count for common taxa is higher, but regional unique taxa maintain a consistent median value.

Finally, we identified taxa that distinguish between PD and HC with many findings supported by existing literature (Supplementary Table S2). For example, *Citrobacter rodentium* has been experimented in multiple studies, where intestinal infection with this pathogen is shown to impair dopamine metabolism and induce PD-like pathology in mice model (Cannon *et al.* 2020, He *et al.* 2024). Our model also identified multiple pathogens belonging to the Stenotrophomonas genus as important taxa, including *Stenotrophomonas maltophilia*, *Stenotrophomonas rhizophila*, *Stenotrophomonas pictorum*. This aligns with the literature that the presence of the *Stenotrophomonas* genus is found to be elevated in patients with PD and also in patients with rapid eye movement sleep behavior disorder, a condition closely associated with PD (Zhang *et al.* 2024). As another example, the model identified Acinetobacter courvalinii and *Acinetobacter johnsonii* as important taxa. This also corroborates with the literature, where the *Acinetobacter* genus is found to be elevated in PD compared to HC (Li *et al.* 2017).

### 3.6 Computational efficiency of IMPACT

We systematically checked both training and inference time for different input data sizes from 200 to 1200 samples. Each setting was repeated five times and the results are shown in Supplementary Fig. S10. All our experiments were based on a single NVIDIA RTX A5000 GPU. For a 1000-sample dataset, the training process of IMPACT generally costs less than two and half minutes and the inference process was around one second. The speed would be much faster if IMPACT is deployed on a modern GPU device.

## 4 Discussion

In this study, we have introduced IMPACT, an interpretable deep learning approach for microbiome data analysis. IMPACT reimagines three recently introduced concepts in deep learning for microbiome data in a single feature engineering step which produces CNN ready images based on low-dimensional representations of a 1D taxa vector based on their similarity. As such enables interpretable classification of disease outcomes using gut microbiome data. IMPACT refines taxa similarity measures by integrating each taxa’s associated metabolites into a two-dimensional representation where taxa in similar image regions indicate biological similarity.

Taxonomic approaches are a promising measure for taxa similarity as a proxy to determining similar outcomes for disease. However, some species of bacteria such as *Escherichia coli* can be pathogenic or harmless toward humans depending on their strain (Ingerson-Mahar and Reid 2011). Phylum stratification seeks to exploit the inherent correlation between OTU’s due to their hierarchical taxonomic structure to extract similar information simultaneously. Phylum is an extremely broad grouping and by grouping at Phylum level, we inherently assume that taxa in the same phylum contribute similarly toward disease which has weak biological evidence (Gerhardt and Mohajeri 2018, Magne *et al.* 2020).

The obvious solution would be to consider more high-level taxonomic ranks such as family or class, taxa stratified at these levels should be more similar functionally. This means we would train individual models on each stratified phylogenetic group which could reach into the tens to hundreds depending on the chosen level. This approach is computationally inefficient given an individual model is trained for each stratified group. A key benefit of CNN’s is their ability to learn important features from high-dimensional input, by stratifying taxa into small groups the CNN cannot consider spatial relationships between features of different groups which may reduce the amount of information input into the model for it to learn patterns in the data. We consider taxa similarity based on their functioning in human cometabolism by considering the metabolic potential of taxa through their ability to produce and transform metabolites from information in the Agora2 database.

Complex ML models, including DL models are often referred to as “Black Boxes” due to their lack of interpretability. However, interpretability is crucial in biomedical and bioinformatics applications given the need to understand biological features leading to predictive outcomes. Our workflow allows not only for identification of important taxa for phenotype prediction but also allows for downstream analysis of functional microbial features of important taxa by integrating annotated databases. Furthermore, this method can be used for binary and multiclass classification problems. Target labels can be customized so features important to the specific classification problem of interest can be inspected. By manipulating the target labels, the model can provide feature importance for any classification problem, allowing the user to contrast and compare important taxa for their specific problem of interest.

There are certain limitations and assumptions in our approach that warrant further investigation. While we have focused on gut microbiome data in this study, future research could expand the applicability of IMPACT to other microbiome sites in the human body. Additionally, incorporating more environmental variables, such as ethnicity, smoking status, dietary habits, and medication, could enhance the performance of the model, providing a more comprehensive understanding of the factors influencing disease prediction. Moreover, the performance of IMPACT is based primarily on extremely limited data set sample sizes, traditionally we expect larger amounts of training data though this a common problem in bioinformatics with no single simple solution.

In summary, IMPACT provides new ways to investigate phenotype classification results using functional microbial databases. Currently, there are not many of these databases and finding a single database that contains annotations for all taxa that you encounter in a dataset is very unlikely. As more microbiome data and functional annotation of taxa become available, we expect IMPACT to become increasingly effective in handling and explaining diverse data, ultimately benefiting various applications in human health and disease prediction.

## Supplementary Material

btae702_Supplementary_Data

## Data Availability

*Data*: Parkinson Disease dataset is available at https://sydneybiox.github.io/PD16SData; American Gut Dataset can be obtained from https://qiita.ucsd.edu/study/description/10469 and is also made available on Figshare at https://doi.org/10.6084/m9.figshare.26805253. *Code*: https://github.com/SydneyBioX/IMPACT. We provide direct installation of IMPACT via pip.
