# Supplementary material for: IMPACT: interpretable microbial phenotype analysis via microbial characteristic traits
Source: Bioinformatics. 2024 Dec 10;41(1):btae702. doi: 10.1093/bioinformatics/btae702 (PMC11687948; doi:10.1093/bioinformatics/btae702)
Supplement: btae702_Supplementary_Data [file btae702_supplementary_data.docx]

Supplementary Materials

## Supplementary Methods

### **Simulation data**

In our simulation study, we also simulated human gut microbial feature abundances using metaSPARSim [(Patuzzi *et al.*, 2019)](https://paperpile.com/c/CA4Cui/WdKU). MetaSPARSim simulates microbiome count data using a Multivariate Hypergeometric distribution to reflect the sparsity and the composition nature of the microbiome data.

Using metaSPARSim, we generated a total of 36 microbiome simulation datasets, each of which consists of 100 samples with two outcomes group A and group B. Group A was designed to reflect the PD group of the Aho dataset. To achieve this, we estimated the mean intensity of taxa from the PD group of the Aho dataset and used as the reference for group A. We then applied various fold changes to simulate the taxa intensities for group B. Note that, after mapping each taxa to bacterial species using a 90% BLAST percentage identity score threshold, we removed the taxa that did not get mapped to any bacteria species. In total, we generated 2589 taxa for each dataset, except for the datasets under the “number of features” scenario.

We simulated the following scenarios:

1. Sparsity: We simulated various sparsities of 0.58, 0.64, 0.72, 0.78, 0.84, 0.89 and 0.92. This was achieved by first estimating the median library size of the PD group of the Aho data and then multiplying the median library size by varying factors of 1, 2, 5, 10, 0.5, 0.2 and 0.1. A factor > 1 means a larger library size and thus less sparsity. We kept the FC between 1.3 and 2 and the number of DA taxa to 400 (350 after removing non-mapped taxa).
2. Differentially abundant (DA) taxa: We simulated various numbers of DA taxa of 50, 100, 200, 300, 400, 500, 600 and 700. After removing non-mapped taxa, this resulted in a final number of 45, 88, 181, 264, 350, 437, 528 and 611 DA taxa. We kept the FC between 1.3 and 2.
3. Taxa fold change: We simulated various lower and upper bounds of the FC, including (1.1, 1.3), (1.3, 1.5), (1.5, 1.7), (1.7, 2.0), (2.0, 2.5), (2.5, 3.0), (3.0, 3.5), (3.5, 4.0). We kept the number of DA taxa to 400 (350 after removing non-mapped taxa).
4. Number of features: We varied the total number of taxa to 200, 500, 1000, 1500, 2000, 3000, 4000, and 5000. After removing non-mapped taxa, this resulted in a final number of 182, 438, 867, 1293, 1733, 2589, 3439 and 4317 taxa. The FC is kept between 1.3 and 2. The number of DA taxa is kept at 13% of the total number of taxa.
5. Number of samples and class imbalance. We simulated the following number of samples in group A and group B to mimic class imbalance: (10, 90), (20, 80), (30, 70), (40, 60) and (50, 50). We kept the FC between 1.3 and 2 and the number of DA taxa to 400 (350 after removing non-mapped taxa).

### **Data preprocessing**

The collection and preprocessing of all datasets were described in [(Xu *et al.*, 2023)](https://paperpile.com/c/CA4Cui/deSgL). In summary, all data was preprocessed using the DADA2 pipeline[(Callahan *et al.*, 2016)](https://paperpile.com/c/CA4Cui/nAzkh)v1.16) using the silva 138 database as taxonomic reference database for microbiome annotations[(Quast *et al.*, 2013)](https://paperpile.com/c/CA4Cui/uvGd8).

The seven processed datasets are combined into a single FASTA file containing 128,160 unique ASV’s. Next, we obtain species-level resolution by performing Basic Local Alignment Search Tool (BLAST) searches against a 16s Ribosomal RNA database generated by the National Center for Biotechnology Information (NCBI). Here, we used a percentage identity score of 70%, selecting ASV’s with at least 70% of base pairs (bp)match between our ASV sequence and the NCBI reference sequence (Supplementary Figure S2). Consequently, 8,843 ASV’s (6.9%) were excluded from further data analysis.

Based on the species-level resolution obtained from BLAST searches, each ASV was mapped to the corresponding metabolite information in an AGORA2 dataset, which contained functional microbial features for 7,302 species. Note that the raw abundance counts of each ASV was transformed into relative abundance per sample, and samples where the sum of the relative abundance of all ASV’s was not equal to 1 were also removed for accurate data analysis. Lastly, we performed an arcsine transformation on the resulting relative abundance data.

For simulation microbiome datasets, we transformed absolute feature abundance to relative abundance to account for variation in total reads across samples. Then we performed an arcsine transformation on the resulting relative abundance microbiome data.

### **Feature Engineering**

We utilize taxa relative abundance and associated metabolites to produce low dimensional representations of their similarity by converting non-image, vectorized data into a well-organized image form. Inspired by DeepInsight [(Sharma *et al.*, 2019)](https://paperpile.com/c/CA4Cui/C77H), this is implemented through a convex hull algorithm aiming to find the minimum box covering all the sample points after they are embedded into a two-dimensional plane using non-linear dimensionality reduction techniques such as t-distributed stochastic neighbour embedding (t-SNE) [(Zhou and Jin, 2020)](https://paperpile.com/c/CA4Cui/9IXR) and/or uniform manifold approximation and projection(UMAP)[(McInnes *et al.*, 2018)](https://paperpile.com/c/CA4Cui/xCgL).

Specifically, for a dataset containing n patients with relative abundance information of m taxa, the 2D coordinates of features (taxa) after t-SNE or kernel PCA would be:

$\{(x_{1},y_{1}), (x_{2},y_{2}), ..., (x_{m},y_{m})\}$,

where (x,y) represents the embedding plane and each point represents a feature. We then try to find the minimum square box which covers all the points using a convex hull algorithm. For the convenience of feature feeding-in in the following networking training process, we perform rotation to make the square box horizontal. Let two corner points of the square be $(x_{c1},y_{c1}), (x_{c2},y_{c2})$, the rotation angle $\phi$ would be computed by:

$\phi= {tan}^{-1}(\frac{y_{c2}-y_{c1}}{x_{c2}-x_{c1}})$.

Then the mapped pixel location of a feature (taxa) would be simply converted from multiplying the rotation matrix R with its original coordinate, i.e.,

$({px}_{new},{py}_{new}) = R\cdot(x_{old},y_{old})$,

where (px,py) represents the plane of ready-to-use image-form and

$$R=\left[ 1_{-sin\phi cos\phi}^{cos\phi sin\phi} \right]$$

Image size is a hyper-parameter, which is the values of each pixel representing the relative combined abundance values of the taxa mapped to that pixel.

The transformer for two dimensional representations is easily made reproducible for unseen data as it can be saved and loaded through the *pickle* package. This results in a single channel image with similarity between taxa associated with the spatial proximity of the pixels they are assigned to.

### **Model Framework Rationale**

The concept of grouping taxa that provide similar disease outcomes to take advantage of the CNN architecture. The data input for each node contains correlated information and adjacent nodes contain correlated information based on their spatial distance. In short, this introduces relationships between taxa into the model in a way that is congruent to information of pixels being correlated to their spatial proximity for image classification tasks.

A different approach to dimension reduction specifically for microbiome data is introduced by [(Oh and Zhang, 2020)](https://paperpile.com/c/CA4Cui/8zyd) in DeepMicro. This is a two-stage process which first uses the encoder network from an Autoencoders (AE) for dimension reduction of microbiome data and then trains classical machine learning models on the dimension reduced output of the encoder. However, determining feature importance through this approach is problematic given the dimension reduction done by the encoder is not interpretable with the decoder.

Microbiome data by nature is high dimensional, this can be controlled by choosing which taxonomic rank to use as features, the broader the taxonomic rank the fewer features you have. Dimension reduction techniques like t-SNE [(Zhou and Jin, 2020)](https://paperpile.com/c/CA4Cui/9IXR) and UMAP[(McInnes *et al.*, 2018)](https://paperpile.com/c/CA4Cui/xCgL) are used in many omics fields with innate high dimensional data like Single Cell RNA [(Feng *et al.*, 2020; Lin *et al.*, 2019)](https://paperpile.com/c/CA4Cui/ZkRD+Hvkv) to produce low dimensional representations of complex high dimensional data. The motivation of this is to find features or samples that are similar and cluster them in a lower dimensional space.

The case for a tabular to image data transformation rests mainly in using a CNN architecture for phenotype prediction. While CNN’s can be applied to 1D input vectors using an appropriate kernel shape for phenotype prediction as shown in [(Sharma *et al.*, 2020; Sharma and Xu, 2021)](https://paperpile.com/c/CA4Cui/B4GJ+RKRt), this approach does not fully utilize the strength of CNN’s which can consider relationships between inputs in a multi-dimensional space. This drawback becomes readily apparent when integrating taxa similarity measures given we will be restrained to considering relationships between taxa represented by their arrangement in only one dimension.

### **Model Architecture**

An important aspect of our model is an emphasis on extracting the most accurate representation of which areas of sample images lead to a classification result. To do this we utilize a residual block architecture which has shown to be especially efficient for training generalizable networks[(He *et al.*, 2020)](https://paperpile.com/c/CA4Cui/wZwe) which is key given the small sample size of most microbiome datasets.

The residue connection of the layer l in the network could be represented as the sum of an identity branch and a perceptron branch, i.e.,

$y_{l}= x_{l+1}= x_{l} + F(x_{l}, W_{l})$.

After recursive computation, we get the formula of a general deeper layer L as:

$x_{L}= x_{l}+\sum_{i=l}^{L-1} 1F(x_{i}, W_{i})$.

The backpropagation process of this block would be:

$\frac{\partial loss}{\partial x_{l}}=\frac{\partial loss}{\partial x_{L}}\frac{\partial x_{L}}{\partial x_{l}}=\frac{\partial loss}{\partial x_{L}}(1+\frac{\partial}{\partial x_{l}}\sum_{i=l}^{L-1} 1F(x_{i}, W_{i}))$,

where loss represents the objective function. This makes the chain derivative formula change from derivative multiplication to addition and since $\frac{\partial}{\partial x_{l}}\sum_{i=l}^{L-1} 1F(x_{i}, W_{i})$=-1 is an event with extremely low probability, thus residue connection prevents gradient problems during small-dataset-training.

To increase the model's ability to capture the spatial feature importance, we integrate spatial attention [(Sanghyun Woo, Jongchan Park, Joon-Young Lee, In So Kweon)](https://paperpile.com/c/CA4Cui/8uzU) into each residual block to facilitate interpretable feature extraction by saliency maps. This block could be implemented as a two-step-attention, i.e., channel attention followed by spatial attention:

$output={{Attention}_{spatial}\{Attention}_{channel}(input)\otimes input\}\otimes{\{Attention}_{channel}(input)\otimes input\}$

, where

$${Attention}_{spatial}(input)=\sigma(f^{k\times k}([AveragePool(input);MaxPool(input)]));$$

and

$${Attention}_{channel}(input =\sigma(MLP(AveragePool(input))+MLP(MaxPool(input)) ).$$

$f^{k\times k}$ means convolutional kernel with size k, and MLP means general multi-layer perceptron.

As to our network architecture, each residual block contains two convolutional layers, with a sequential layer structure as follows:

- Convolutional Layer
- Batch-normalization
- Activation function
- Convolutional Layer
- Batch-normalization
- Spatial attention map
- Identity function
- Activation function

This architecture follows the internal sequential structure of residual blocks introduced by the original authors in *Deep Residual Learning for Image Recognition*[*(He et al., 2015)*](https://paperpile.com/c/CA4Cui/cuFF)*.* Our model allows the user to choose the number of filters, filter size and stride of each residual block explicitly using a simple list structure.

####

For classification tasks, this methodology utilizes the gradients of classification scores with respect to convolutional layers feature maps to identify the parts of the input image that contribute most to the final classification scores. Typically Grad-CAM is used to identify which regions of a single sample image lead to a particular classification outcome.

This differs to the pseudo images as all images have the same taxa mapped to the same pixel in every image, the benefit of this is that we can investigate the regions of images which leads to different label classification outcomes across all samples rather than just a single sample to determine which Taxon contributed most to each classification label. Once regions that represent importance have been determined, we can translate this to feature importance based on which features are mapped to regions with high importance.

To summarize, our inputs are pseudo images in which taxa are mapped to the same pixel in every image. The model learns the regions of an image that contribute to a classification outcome using spatial attention transformers during the training process. We then utilize an existing method, that is, Grad-CAM to extract scores for which pixels in an image contribute to a particular classification outcome. We repeat the above process for all images and determine which regions of an image are the most important for a particular class outcome by averaging importance scores for each pixel across images from that class. The most important pixels can then be mapped back to the taxa associated with this pixel to identify the features most important for classification of a class. Once we have extracted important taxa, we can then fully utilize all of the annotations provided in the microbial databases to investigate which classification outcomes are associated to a particular metabolite. In the results section, we selected a subset of PD and HC patients as an example to illustrate the selection of important taxa in the classification of PD and HC and their literature evidence.

### **Performance and evaluation**

To compare predictive performance of the model, all seven Parkinson’s disease datasets from four different regions were used together as a single dataset. We considered two different classification problems across three evaluation schemes:

[A] Binary classification of Parkinson’s disease status using cross validation on all datasets;

[B] Multi-class classification problem predicting both the Parkinson’s disease status and the country of origin for the sample.

[C] Binary classification of Parkinson’s Disease on independent test sets

We compare models specially designed for microbiome as well as classical machine learning. These are:

[1] TaxoNN using the best-performing architecture as stated in the original paper;

[2] Support Vector Machine;

[3] Random Forest

[4] DeepMicro (Using code provided by authors, only supports Binary classification)

In Models [2] and [3] a grid search was performed on a random subset of the total data for optimal hyperparameter selection. DeepMicro results were obtained using the methods proposed in the original paper using the code provided by the authors, as such the methodology used is slightly different to the rest of the models presented and is thoroughly described in the original paper’s results section. All random splitting was performed using a stratified split method to prevent imbalanced training datasets.

### **Evaluation metrics**

All models in evaluation schemes [A] and [B], were evaluated based on a ten times repeated five-fold cross-validation method. Here the data was split into a temporary dataset containing four folds and a testing dataset containing one fold. The temporary dataset was then split into training and validation by a 75/25 ratio split amounting to a 60:20:20 split of the original dataset into the respective training, testing and validation sets. The validation set was used to determine the best-performing weights and biases for IMPACT and TaxoNN models. The SVM and RF classification models were fit on the training set and predictions were made on the testing set thus the validation set is not used in the training process for these models.

For the evaluation scheme [C] we initially split the dataset to remove all Jin samples such that the training dataset and testing set (Jin) are separate. The training dataset is then randomly split into a training and validation set by a ratio of 75:25. The validation set was used to determine the best-performing weights and biases for IMPACT and TaxoNN models. The SVM and RF models were trained only on the training and not the validation set. The models were then tested on the Jin dataset. This process was repeated ten times to introduce variability in the training dataset.

To evaluate the effect of the stability of our model on stochastic representations of features we have tested different values for parameters controlling the local two-dimensional representation balance between preserving local and global relationships between the data. This is perplexity for TSNE and the number of neighbours for UMAP. The data was split into a training and testing set which was kept constant throughout the comparison of different parameter values. The same training data was repeatedly transformed into image representation using different parameter values, after which a model was trained on the training set and tested on the testing set. For each parameter value, we have repeated the calculation 5 times to observe variability not only between changes from external parameters in emphasizing local versus global similarity of taxa, but also to account for possible variation in performance introduced by the randomness of the projections that are produced by dimension reduction techniques.

**Reference**

[Callahan,B.J. *et al.* (2016) DADA2: High-resolution sample inference from Illumina amplicon data. *Nat. Methods*, **13**, 581–583.](http://paperpile.com/b/CA4Cui/nAzkh)

[Feng,C. *et al.* (2020) Dimension Reduction and Clustering Models for Single-Cell RNA Sequencing Data: A Comparative Study. *Int. J. Mol. Sci.*, **21**.](http://paperpile.com/b/CA4Cui/ZkRD)

[He,F. *et al.* (2020) Why ResNet Works? Residuals Generalize. *IEEE Trans Neural Netw Learn Syst*, **31**, 5349–5362.](http://paperpile.com/b/CA4Cui/wZwe)

[He,Y. *et al.* (2018) Regional variation limits applications of healthy gut microbiome reference ranges and disease models. *Nat. Med.*, **24**, 1532–1535.](http://paperpile.com/b/CA4Cui/UeInX)

[Lin,Y. *et al.* (2019) scMerge leverages factor analysis, stable expression, and pseudoreplication to merge multiple single-cell RNA-seq datasets. *Proc. Natl. Acad. Sci. U. S. A.*, **116**, 9775–9784.](http://paperpile.com/b/CA4Cui/Hvkv)

[McInnes,L. *et al.* (2018) UMAP: Uniform Manifold Approximation and Projection for Dimension Reduction. *arXiv [stat.ML]*.](http://paperpile.com/b/CA4Cui/xCgL)

[Oh,M. and Zhang,L. (2020) DeepMicro: deep representation learning for disease prediction based on microbiome data. *Sci. Rep.*, **10**, 6026.](http://paperpile.com/b/CA4Cui/8zyd)

[Patuzzi,I. *et al.* (2019) metaSPARSim: a 16S rRNA gene sequencing count data simulator. *BMC Bioinformatics*, **20**, 416.](http://paperpile.com/b/CA4Cui/WdKU)

[Quast,C. *et al.* (2013) The SILVA ribosomal RNA gene database project: improved data processing and web-based tools. *Nucleic Acids Res.*, **41**, D590–6.](http://paperpile.com/b/CA4Cui/uvGd8)

[Sanghyun Woo, Jongchan Park, Joon-Young Lee, In So Kweon CBAM: Convolutional Block Attention Module. Korea Advanced Institute of Science and Technology, Daejeon, Korea, pp. 3–19.](http://paperpile.com/b/CA4Cui/8uzU)

[Sharma,A. *et al.* (2019) DeepInsight: A methodology to transform a non-image data to an image for convolution neural network architecture. *Sci. Rep.*, **9**, 11399.](http://paperpile.com/b/CA4Cui/C77H)

[Sharma,D. *et al.* (2020) TaxoNN: ensemble of neural networks on stratified microbiome data for disease prediction. *Bioinformatics*, **36**, 4544–4550.](http://paperpile.com/b/CA4Cui/B4GJ)

[Sharma,D. and Xu,W. (2021) phyLoSTM: a novel deep learning model on disease prediction from longitudinal microbiome data. *Bioinformatics*, **37**, 3707–3714.](http://paperpile.com/b/CA4Cui/RKRt)

[Xu,X. *et al.* (2023) NEMoE: a nutrition aware regularized mixture of experts model to identify heterogeneous diet-microbiome-host health interactions. *Microbiome*, **11**, 51.](http://paperpile.com/b/CA4Cui/deSgL)

[Zhou,B. and Jin,W. (2020) Visualization of Single Cell RNA-Seq Data Using t-SNE in R. In, Kidder,B.L. (ed), *Stem Cell Transcriptional Networks: Methods and Protocols*. Springer US, New York, NY, pp. 159–167.](http://paperpile.com/b/CA4Cui/9IXR)

## Supplementary Figures


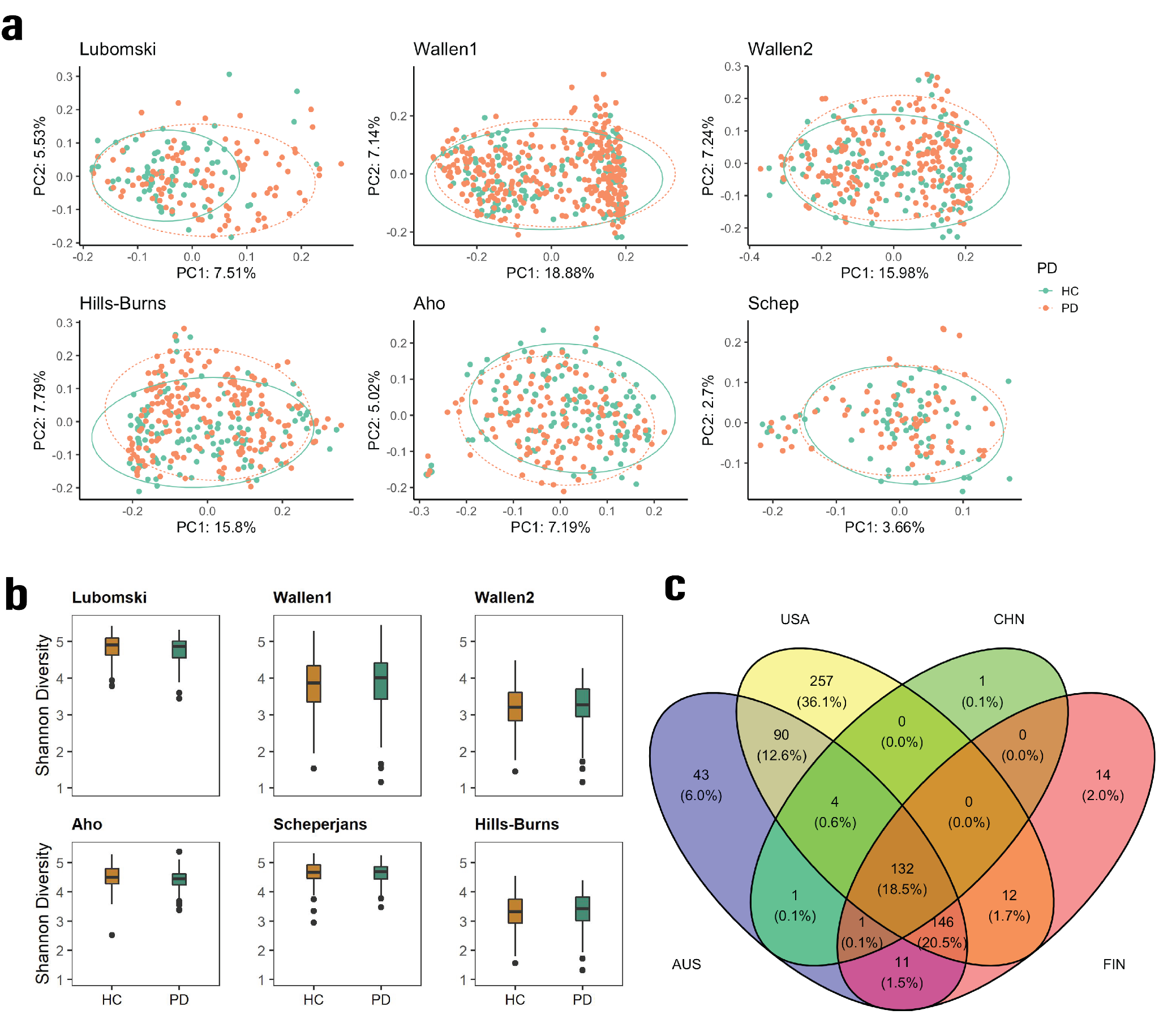


**Supplementary Figure S1.** Exploratory analysis of the PD and HC datasets.

a. PCoA plot showing samples by first two PC on scatterplot based on disease status and 6 data sets. b. Shannon Diversity Index by disease status for 6 datasets. c. Overlap of common species for each dataset by region


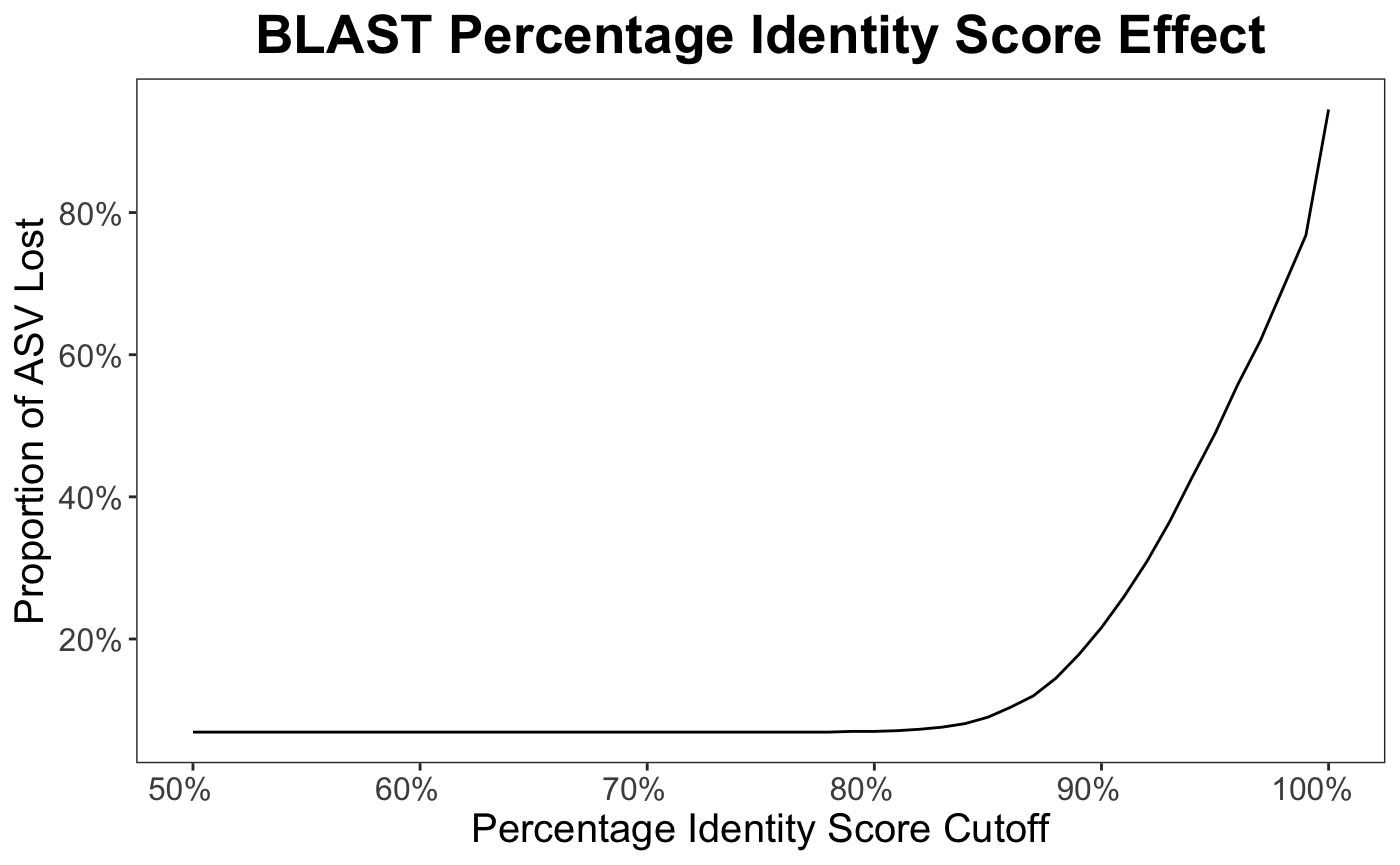


**Supplementary Figure S2.** The effect of BLAST percentage identity scores on the proportion of ASV excluded from our data analysis.

As the cutoff percentage identity score increases, the proportion of ASV’s that are not searchable in the NCBI 16s Ribosomal RNA database increases. In our data analysis, we used a percentage identity score of 70%.


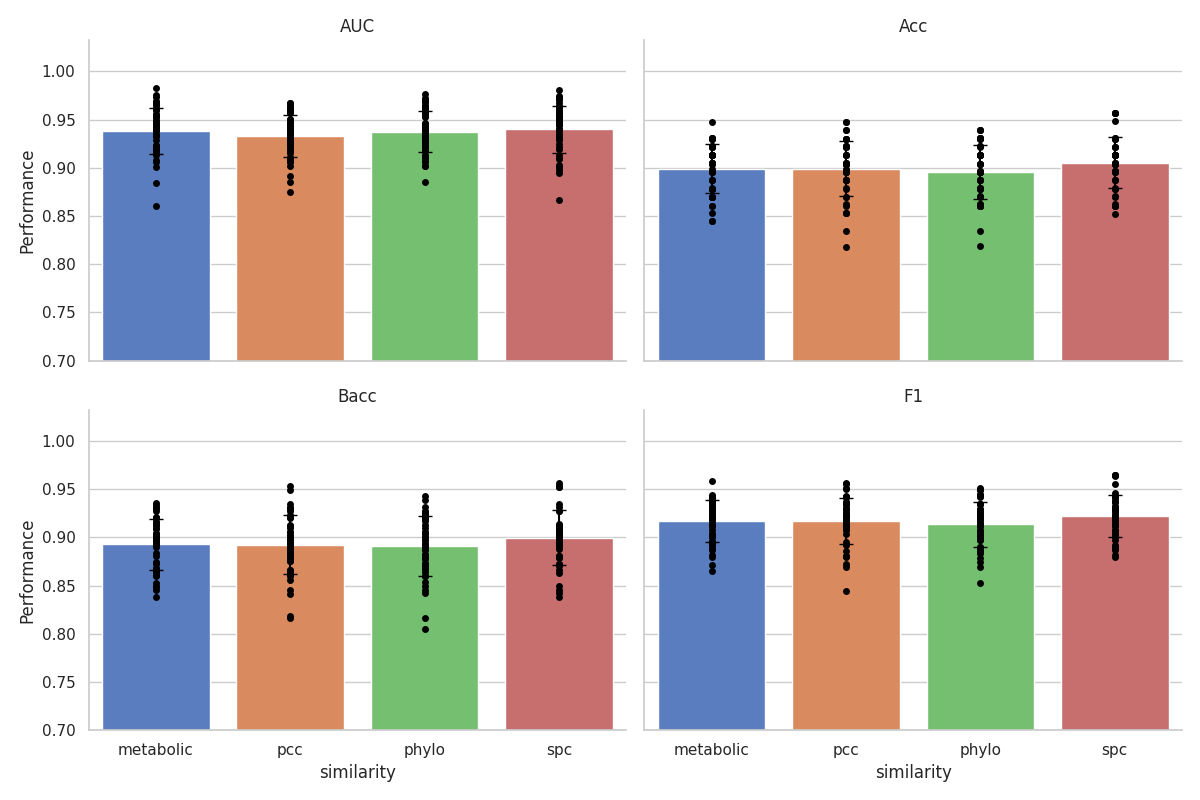


**Supplementary Figure S3.** Bar plots of IMPACT performance when using different similarity construction approaches. Blue represents metabolic outputs, orange for Pearson Correlation, green for phylogeny-based similarity and red for Spearman Correlation. Among them, only metabolic outputs could be directly achieved from public databases without additional preparation and computation. IMPACT shows its robustness on different similarity construction methods.


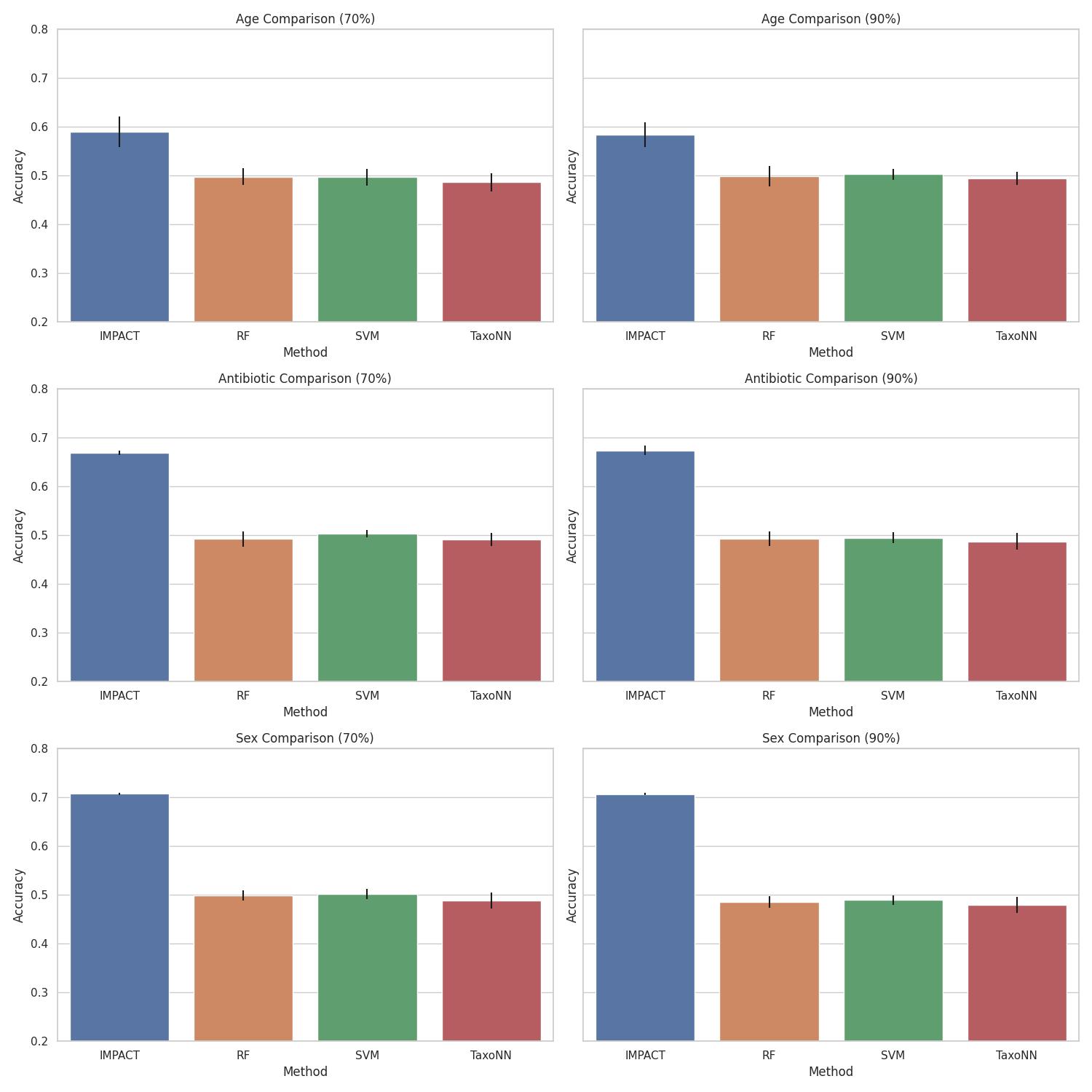


**Supplementary Figure S4.** Bar plots of IMPACT performance when predicting various outcomes by AmericanGut database using different BLAST threshold settings.


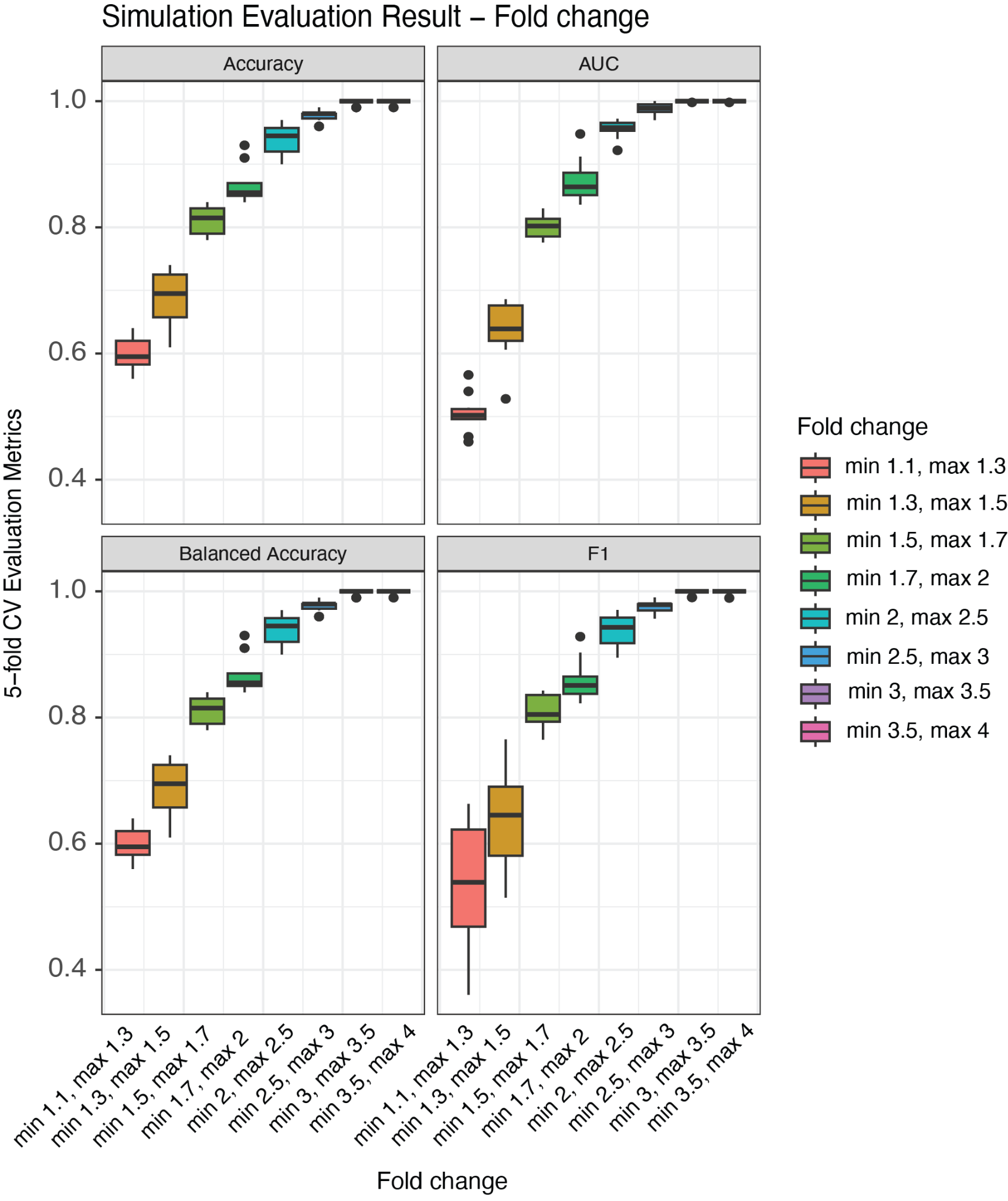


**Supplementary Figure S5.** Evaluation result on varying fold change by metaSPARSim.

We vary the fold change while keeping the number of differentially abundant taxa to 400 and the library size to be the median library size of the reference data (see Methods). 50 samples are simulated for each condition.


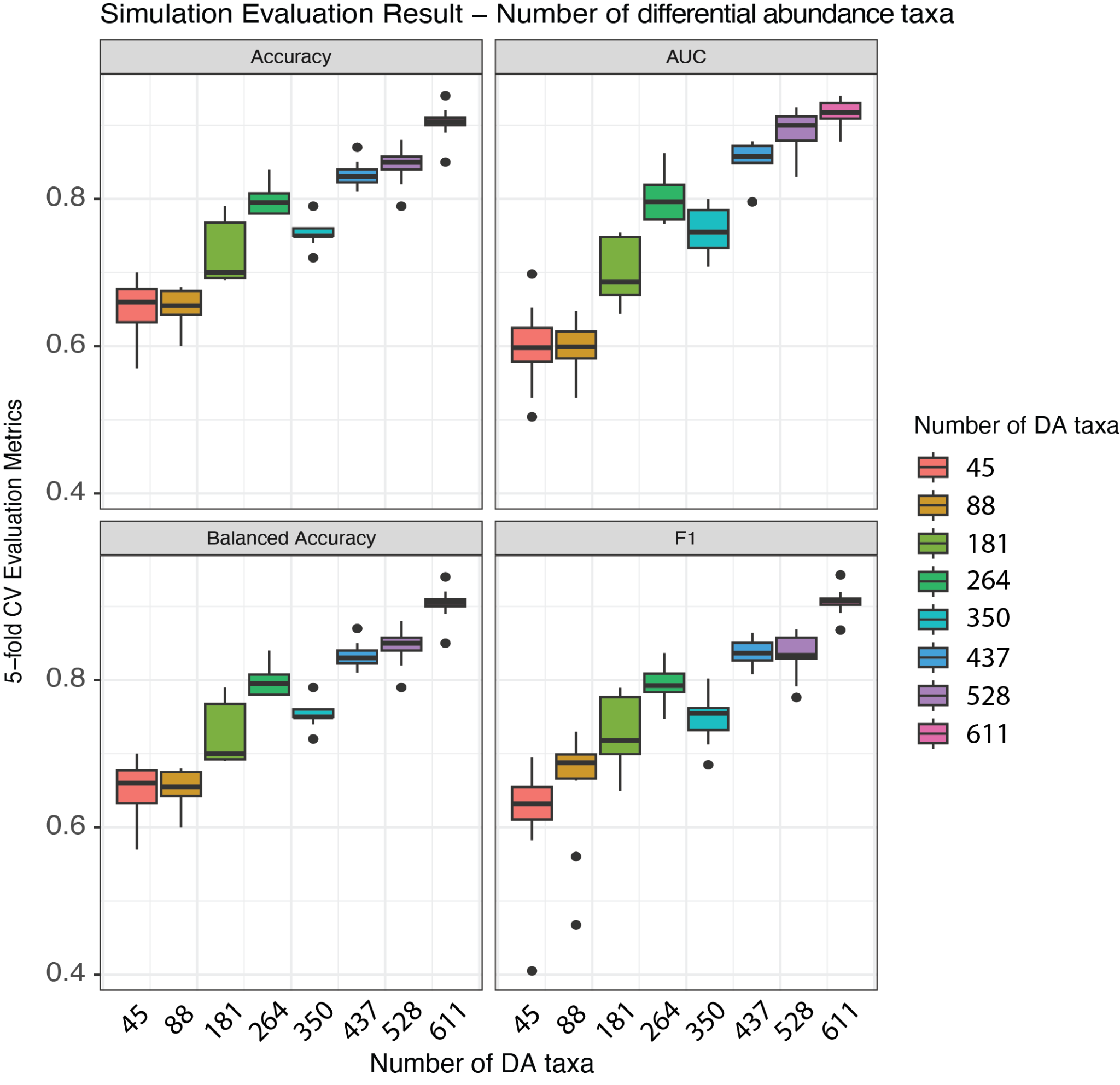


**Supplementary Figure S6.** Evaluation result on varying numbers of differentially abundant taxa by metaSPARSim.

We vary the number of differentially abundant (DA) taxa while keeping the fold change to 1.3 to 2 and library size to be the median library size of the reference data (see Methods). 50 samples are simulated for each condition.


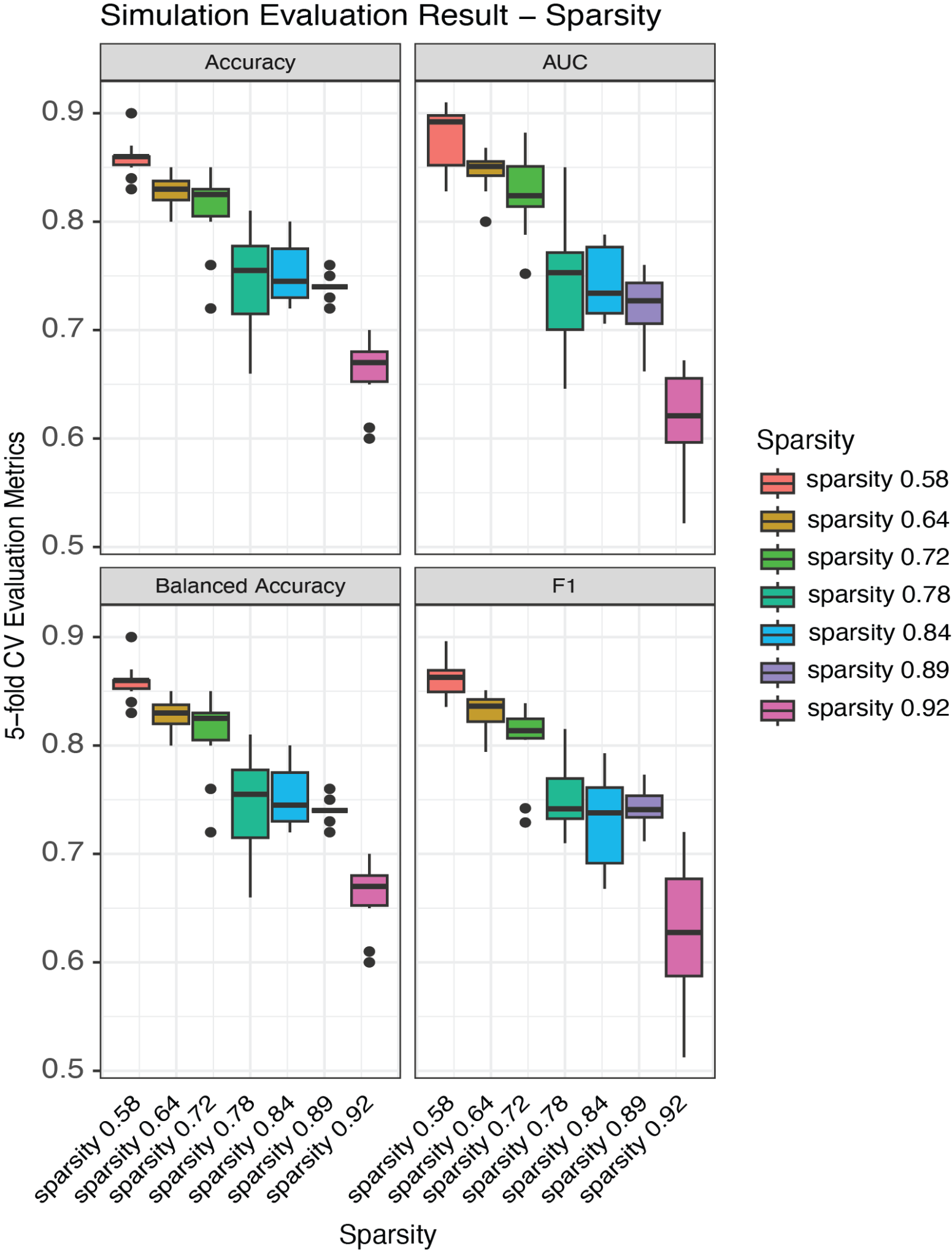


**Supplementary Figure S7.** Evaluation result on varying sparsity by metaSPARSim.

We multiplied the median library size estimated from the reference data by a certain factor, resulting in varying sparsity of the data (see Methods). A smaller library size results in greater sparsity and a larger library size results in less sparsity. The number of differentially abundant taxa is kept at 400. The fold change is kept at 1.3 to 2. 50 samples are simulated for each condition.


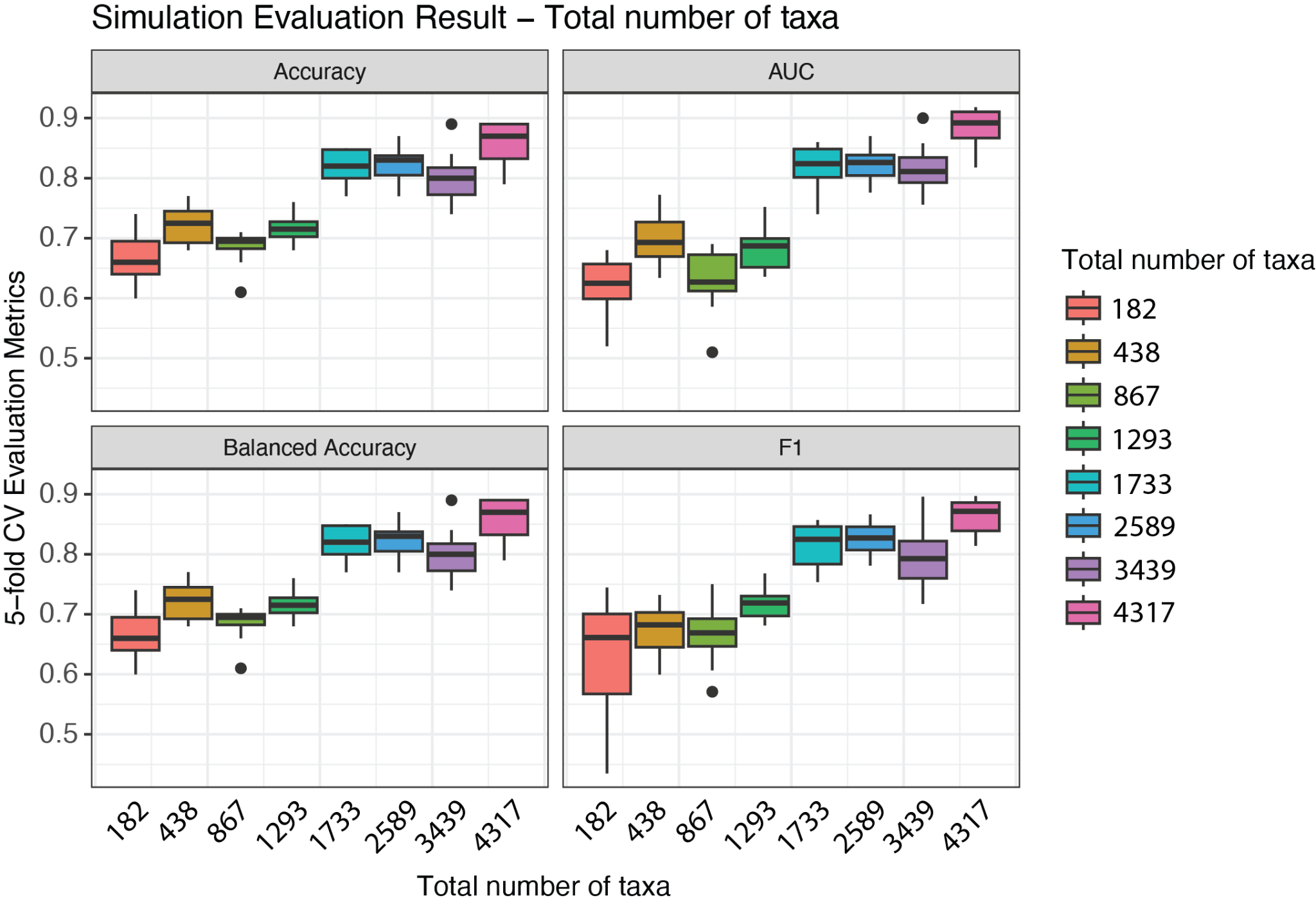


**Supplementary Figure S8.** Evaluation result on varying total number of taxa by metaSPARSim.

We varied the number total number of taxa while keeping the proportion of DA taxa to be 13% of the total number of taxa. The fold change is kept at 1.3 to 2. 50 samples are simulated for each condition.


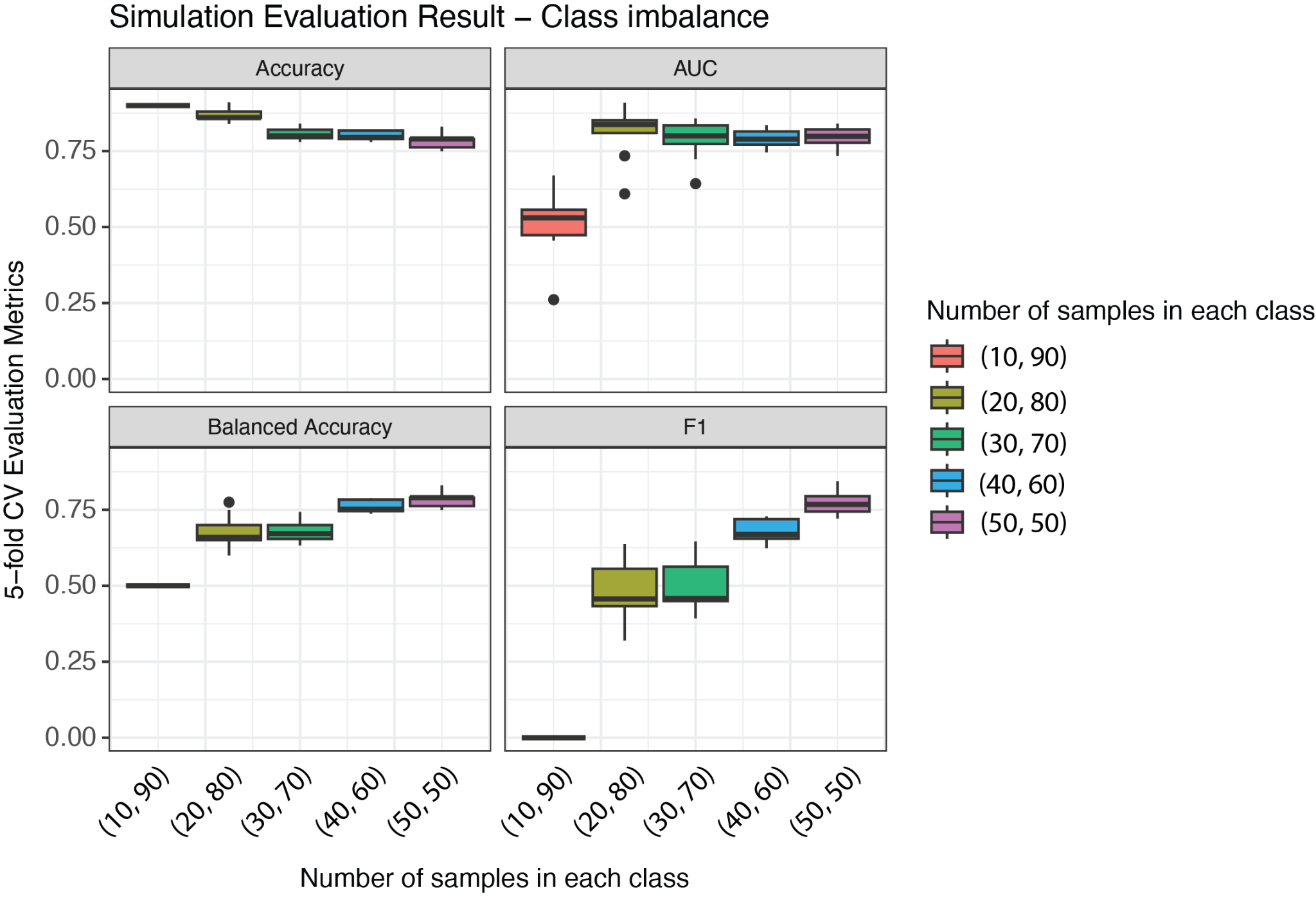


**Supplementary Figure S9.** Evaluation result on varying class imbalance by metaSPARSim.

We varied the number of samples in the two sample groups, resulting in varying degrees of class imbalance. The number of differentially abundant taxa is kept at 400. The fold change is kept at 1.3 to 2.

###
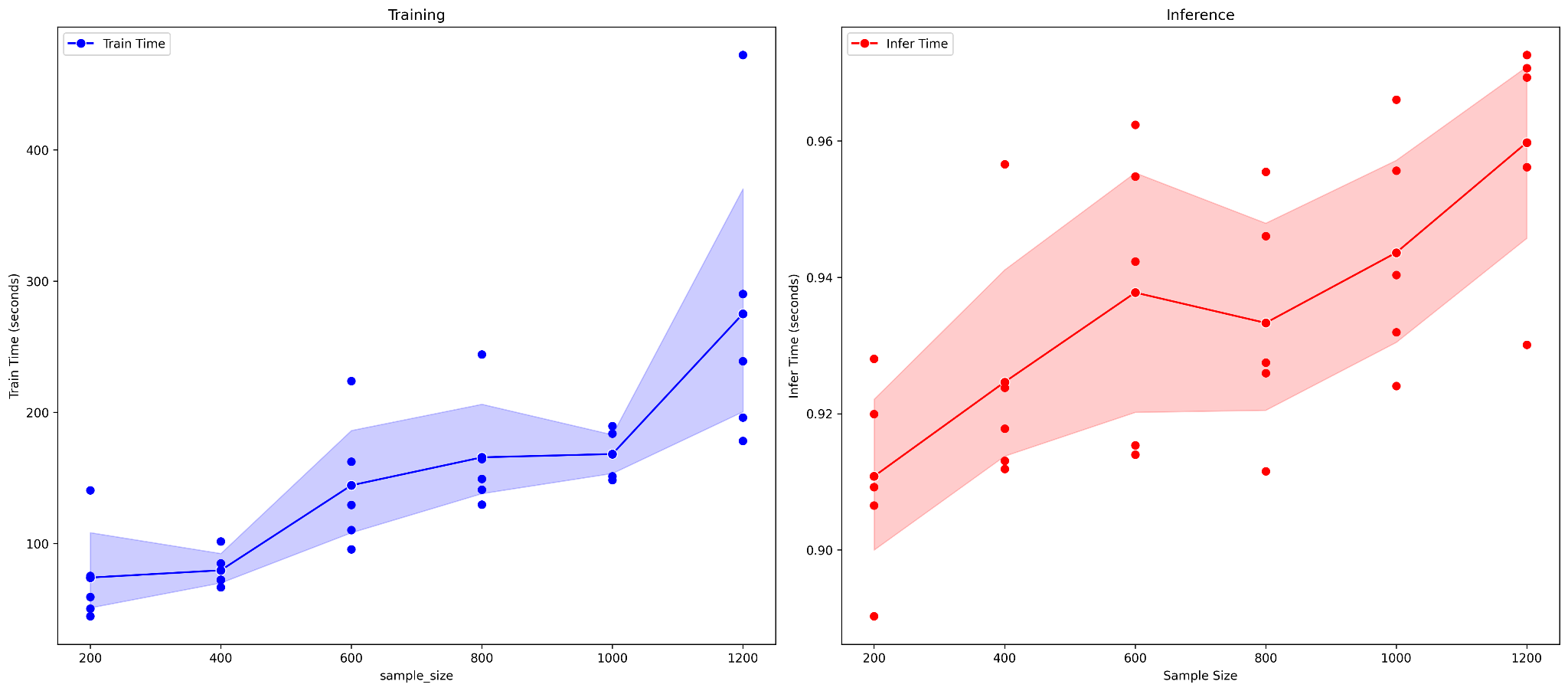


**Supplementary Figure S10.** Computational time of IMPACT when training (blue) or inferring (red) on different input data sizes. Data size from 200 to 1200, each setting was repeated 5 times (dots represent each repeat while ribbons represent statistical error range).

##

## Supplementary Table

### Supplementary Table S1

Table showing the collection of the datasets used in this manuscript. The data has been downloaded from https://sydneybiox.github.io/PD16SData.

| Index | Study ID | Cohort | Sample Size | Sampling | DNA Extraction | 16s Region | ENA Accession |
| --- | --- | --- | --- | --- | --- | --- | --- |
| 1 | Lubomski | AUS | 74PD,74HC | Home Collection, stored at -80 ℃ | MP Biomdeical FastDNATM SPIN Kit | V3-V4 | PRJNA808166 |
| 2 | Wallen1 | USA | 323PD, 184HC | Home Collection, stored at -20 ℃ | MoBio PowerSoilDNA Isolation Kit | V4 | PRJNA601994 |
| 3 | Wallen2 | USA | 197PD,130HC | Swabs, delivered at RT | MoBio PowerMag Soil Kit | V4 | PRJNA601994 |
| 4 | Hills-Burns | USA | 196PD,130HC | Swabs, delivered at RT | MoBio PowerMag Soil Ki | V4 | PRJNA601994 |
| 5 | Aho | FIN | 64PD,64HC | Home Collection, DNA Stabilizers and stored in Fridge | PSP-Spin Stool Kit | V3-V4 | PRJEB27564 |
| 6 | Scheperjans | FIN | 72PD,72HC | Home Collection, DNA Stabilizers and stored in Fridge | PSP-Spin Stool Kit | V1-V3 | PRJEB4927 |
| 7 | Jin | CHN | 72PD,68HC | NA | NA | V3-V4 | PRJEB588834 |

### Supplementary Table S2

Using 2,294 PD and HC samples, we identified the following 37 taxa as important taxa in distinguishing PD and HC.

| Important taxa selected for PD | P_Proteobacteria_Brevundimonaspondensis,  P_Proteobacteria_Achromobacteragilis,  P_Proteobacteria_Brevundimonasvesicularis,  P_Proteobacteria_Acidovoraxvalerianellae,  P_Proteobacteria_Delftiaacidovorans,  P_Proteobacteria_Rhizobiumflavescens,  P_Proteobacteria_Shinellacurvata,  P_Proteobacteria_Stenotrophomonasmaltophilia, P_Proteobacteria_SphingopyxisalaskensisRB2256, P_Proteobacteria_Stenotrophomonasrhizophila, P_Proteobacteria_Acinetobactercourvalinii,  P_Proteobacteria_Pseudomonasalloputida,  P_Proteobacteria_Acidovoraxwautersii,  P_Proteobacteria_Variovoraxparadoxus,  P_Proteobacteria_Aquabacteriumcommune,  P_Proteobacteria_Acinetobacterjohnsonii,  P_Proteobacteria_Acidovoraxanthurii,  P_Proteobacteria_Brevundimonasbullata, P_Proteobacteria_BrevundimonassubvibrioidesATCC15264, P_Proteobacteria_Caulobacterflavus,  P_Proteobacteria_MethylorubrumpopuliBJ001, P_Proteobacteria_MethyloversatilisuniversalisFAM5,  P_Proteobacteria_Nevskialacus,  P_Proteobacteria_Nevskiaramosa,  P_Proteobacteria_Pseudoxanthomonasicgebensis, P_Proteobacteria_Sphingopyxissolisilvae,  P_Proteobacteria_Thermomonascarbonis,  P_Proteobacteria_Sphingobiumxenophagum,  P_Proteobacteria_Sphingobiumlimneticum,  P_Proteobacteria_Agrobacteriumarsenijevicii,  P_Proteobacteria_Brevundimonasdiminuta,  P_Proteobacteria_Citrobacterfreundii, P_Proteobacteria_StenotrophomonaspictorumJCM9942, P_Proteobacteria_Sphingomonaskoreensis, P_Proteobacteria_Novosphingobiumchloroacetimidivorans, P_Proteobacteria_Pseudoxanthomonasjaponensis,  P_Proteobacteria_Mesorhizobiumterrae |
| --- | --- |
